# Plant-Derived Peptide–Polymer Therapeutics for Cutaneous Infections and Inflammation: Mechanistic Basis, Delivery Design and Translational Considerations

**DOI:** 10.3390/pharmaceutics18060729

**Published:** 2026-06-12

**Authors:** Adnan Amin, Mozaniel Santana de Oliveira, Touseef Nawaz, Oberdan Oliveira Ferreira

**Affiliations:** 1 Department of Life Sciences, Yeungnam University, Gyeongsan 38541, Republic of Korea; 2 Laboratory of Pharmacology of Inflammation and Behavior, Graduate Program in Pharmaceutical Sciences, Institute of Health Sciences, Federal University of Pará, Belém 66075-110, Brazil; mozaniel@ufpa.br; 3 Department of Pharmacy, Qurtuba University of Science and Information Technology, Peshawar 25100, Pakistan; 4 Program in Biotechnology, Federal University of Pará (UFPA), Belém 66075-110, Brazil

**Keywords:** hevein-like peptides, PEGylation, cutaneous infections, translational outcomes, wound healing

## Abstract

Cutaneous infections and chronic inflammatory wounds remain difficult to treat because antimicrobial resistance, polymicrobial biofilms, excessive protease activity, oxidative stress, and impaired barrier repair collectively reduce the effectiveness of conventional topical therapies. Plant-derived antimicrobial peptides (AMPs) and peptide-associated bioactives offer antimicrobial, antibiofilm, immunomodulatory, and tissue reparative potential; however, their clinical translation is limited by proteolytic instability, poor stratum corneum penetration, short cutaneous residence time, formulation variability, cytotoxicity risks and limited human evidence. The key research gap is the lack of an integrated translational framework linking plant-derived peptide bioactivity with polymer engineering, advanced delivery systems, skin microenvironment biology, manufacturability, and regulatory feasibility. This review aims to critically evaluate the design principles, therapeutic mechanisms, delivery platforms, and translational barriers of plant-based peptide–polymer therapeutics for cutaneous infection and inflammation. We summarize major classes of plant-derived antimicrobial peptides, including defensins, cyclotides, thionins, hevein-like peptides, snakins, lipid transfer proteins, and knottin-type scaffolds, and examine engineering strategies such as self-assembly, aromatic N-capping, PEGylation, lipidation, dendritic architectures, and stimuli-responsive conjugation. We further discuss topical matrices, nanocarriers, liposomes, electrospun fibers, and surface-tethered biomaterials as delivery platforms for improving peptide stability, local retention, and controlled release. Finally, we identify key translational bottlenecks, including selectivity, toxicity, scalability, batch reproducibility, regulatory classification, and insufficient clinical validation. Mechanism-driven peptide optimization, quality-by-design manufacturing, standardized preclinical models, and controlled clinical trials will be essential for advancing these systems toward safe and effective dermatological therapies.

## 1. Introduction

Skin infections and inflammation, including both acute and chronic lesions (diabetic foot ulcers and pressure sores), continue to present a significant growing global health issue [1,2]. Chronic wound pathology is identified as being primarily due to arrest and dysregulation of the healing cascade, with continuous presence of neutrophils, elevated levels of reactive oxygen species (ROS), and imbalance of proteases [3,4]. The chronic inflammatory environment results in degradation of the extracellular matrix (ECM) and delayed re-epithelialization [5]. Such wounds frequently support colonization by multidrug-resistant microorganisms. The ESKAPE organisms (*Enterococcus faecium*, *Staphylococcus aureus*, *Klebsiella pneumoniae*, *Acinetobacter baumannii*, *Pseudomonas aeruginosa*, and *Enterobacter* spp.) often have multiple drug-resistant phenotypes that limit the effectiveness of both empirically used and specifically targeted therapies [6,7]. Conventional antimicrobial therapy has become less effective due to the limited development of new antimicrobial drugs and the emergence of adaptive resistance in bacterial populations [8].

Traditional topical therapies are significantly impeded by the intrinsic biophysical barriers of the skin. These include the *stratum corneum*, composed of a ‘brick and mortar’ arrangement of lipophilic keratinized corneocytes embedded in a lipophilic matrix [9,10]. The passage of hydrophilic molecules or macromolecules through the *stratum corneum* is restricted [11]. The delivery of therapeutically effective doses of API directly into the viable tissue layers of the epidermis and dermis without reliance on high-dose application that may produce systemic toxicity and local irritation continues to be a major barrier to successful pharmacotherapy [12]. Plant-derived AMPs are usually short, cationic, amphipathic polypeptides with a range of masses from 2–13 KDa [13]. In general, plant-derived AMPs have wide-ranging antimicrobial effects on microbial membranes by targeting anionic phospholipid components of membranes and the anionic polysaccharide components of lipopolysaccharides and teichoic acids [14]. As such, they can cause rapid disruption of membranes, resulting in pore formation and subsequent microbial lysis [15]. Although most research has focused on the direct antimicrobial effects of plant-derived AMPs, many also have additional roles as modifiers of both the host innate immune system and as degraders of extracellular matrices, which inhibit biofilm formation [16].

The limitations of using native plant-derived AMPs for the treatment of skin wounds may be overcome through the development of biopolymer hydrogel-based composite materials or advanced nanocarrier systems (for example, solid lipid nanoparticles, nanostructured lipid carriers, and electrospun nanofibers) [12]. Such polymeric formulations act as protective systems that prevent enzymatic degradation of peptides while controlling the timing and location of peptide release [13]. When formulated into biopolymer hydrogels, these systems mimic the natural environment by providing a moist, bio-instructive substrate that absorbs excess exudate, enhances gas exchange, and promotes cellular attachment and the angiogenic response necessary for rapid healing [17]. Despite increasing interest in plant-derived AMPs, advanced wound biomaterials, and topical nanocarriers, these areas are often discussed as separate research domains [18]. In chronic wounds, therapeutic failure is driven not only by planktonic infection but also by biofilm-protected microbial communities, protease-rich exudates, oxidative stress, and persistent inflammatory signaling [3,19]. Peptide–polymer systems therefore require an integrated design logic that connects antimicrobial potency with protease protection, skin barrier penetration, local retention, controlled release, host-cell compatibility, and manufacturability [20]. A major research gap remains the absence of a translational framework linking plant peptide structure, polymer engineering, cutaneous delivery, wound microenvironment biology, regulatory feasibility, and clinical validation.

The core objective of this review is to critically evaluate plant-derived peptide–polymer therapeutics as integrated platforms for managing cutaneous infections and inflammation. Previous reviews have provided important insights into antimicrobial peptides for wound infection, AMP-based wound dressings, peptide hydrogels, and nanocarrier-assisted peptide delivery [18,21]. However, these studies mainly address AMPs as a broad therapeutic class, or as delivery systems or wound-dressing materials. Specifically, this review examines major classes of plant-derived antimicrobial peptides, peptide engineering and polymer-conjugation strategies, topical and transdermal delivery systems, antimicrobial and antibiofilm mechanisms, immunomodulatory and wound-repair effects, and key translational barriers. By integrating peptide biology, polymer engineering, skin barrier science, and translational pharmacology, this review aims to define design principles required for clinically credible peptide–polymer therapeutics for infected and inflamed skin.

## 2. Literature Search Strategy

The current review manuscript is based on a comprehensive and targeted literature survey of plant-derived peptide–polymer therapeutics for cutaneous infections and inflammation. Relevant studies were identified using Web of Science, Scopus, PubMed/MEDLINE, ScienceDirect, and Google Scholar. English-language publications from 2000 to 2026 were considered, with emphasis on recent studies from the last 6 years (2020–2026), while earlier foundational reports were retained when needed for mechanistic or structural context. Search terms included “plant antimicrobial peptides,” “defensins,” “cyclotides,” “thionins,” “hevein-like peptides,” “snakins,” “peptide-polymers,” “cutaneous infection,” “wound inflammation,” “biofilm,” “hydrogels,” “nanocarriers,” “topical delivery,” “skin barrier,” “peptide stability,” and “clinical translation.” The selection of literature to be included (inclusion criteria) in the manuscript was based on relevance to peptide classes, polymer engineering, cutaneous delivery, antimicrobial and immunomodulatory mechanisms, translational barriers and clinical-development considerations. The studies were excluded if they were unrelated to skin applications, having no relevance to peptide or biomaterial based therapeutics.

To avoid treating peptide classes, polymer engineering, and delivery technologies as separate topics, this review applies a translational evaluation framework. Each strategy is assessed according to five linked criteria: peptide stability in protease-rich wound environments, antimicrobial selectivity over mammalian-cell toxicity, maintenance of local therapeutic exposure, compatibility with reproducible manufacturing, and validation in skin-relevant infection or wound models. This framework enables a more critical distinction between biologically promising peptide scaffolds and clinically credible therapeutic systems.

## 3. Plant-Derived Bioactive Peptides for Skin Applications

### 3.1. Classes and Sources

Plant-derived AMPs represent an extensive array of innate defense molecules [22,23]. These are also defined by their net positive charge, amphipathic structure, and stabilization through disulfide linkages [24]. Plant defensins are ~5 kDa basic peptides that contain a globular alpha-helix and beta-sheet fold that is very tightly stabilized by four conserved disulfide bridges [16,25]. They are found throughout a number of different species, such as wheat and barley, and they demonstrate broad-spectrum microbicidal activity through selective targeting of various components of the microbial plasma membrane [26]. These include the glucosylceramides present on the surface of various fungi [27]. Cyclotides are relatively large macrocyclic peptides, typically consisting of 28 to 37 amino acids, that have been extracted primarily from the Violaceae and Rubiaceae families [28]. A primary characteristic of cyclotides is the formation of a cyclic cystine knot due to the presence of three interlocking disulfide bonds that define their head-to-tail, circularly knotted topology [29]. Cyclotides can be subdivided into several families, i.e., Möbius, bracelet, and trypsin-inhibitor [30].

Thionins represent another important class of plant-derived AMPs. Thionins consist of 45–47 amino acid sequences that are hydrophobic in nature and possess a high level of basicity. In addition to their high levels of basicity, thionins are stabilized through the presence of three to four disulfide bridges [31,32]. They are abundant in the same types of tissue, such as those of the wheat endosperm or *Nicotiana attenuata*. Due to their high levels of amphipathicity, thionins have proven to be extremely effective membrane-disrupting agents with acute toxicity towards both Gram-negative and Gram-positive bacteria, as well as certain pathogenic fungi [16]. Another important class of plant-derived AMPs includes hevein-like peptides that are ~4.7 kDa in molecular mass. Hevein-like peptides employ a chitin-binding domain (SXFGY/SXYGY) that has been shown to be structurally conserved among all members within this family. This domain is employed to specifically target and disrupt the cell wall of fungi [33].

**Figure 1:**
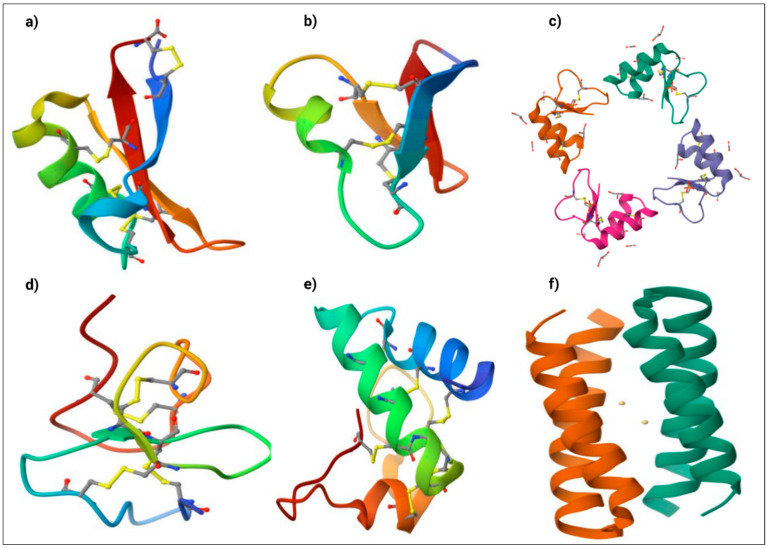
Defensin 1TI5 (pdb_00001ti5) (**a**), cyclotides 7RN3 (pdb_00007rn3) (**b**), thionin 1BHP (pdb_00001bhp) (**c**), hevein-like 6M5C (pdb_00006m5c) (**d**), Snakin-1 5E5Q (pdb_00005e5q) (**e**) and Alpha hairpinins 1Y47 (pdb_00001y47) (**f**). The figure shows representative structures of major plant-derived antimicrobial peptide scaffolds, including defensins, cyclotides, thionins, hevein-like peptides, snakins, and α-hairpinins. These classes differ in cysteine pattern, disulfide topology, backbone organization, and surface charge. Created in BioRender. Zaman, W. (2026). https://BioRender.com/9otxns6. accessed on 31 May 2026.

Snakins (Snakin-1 and Snakin-2) utilize twelve cysteine residues to stabilize their active conformation via six critical disulfide bridges [22]. While their exact mechanism of action remains unknown at this time, it has been demonstrated that snakins do exhibit some level of activity against certain aggressive pathogens. Lipid transfer proteins (LTPs) are small, ~7–10 kDa alpha-helical defense peptides stabilized by four disulfide bridges [34]. Puroindolines are small, highly hydrophobic, and amphipathic peptides that contain a tryptophan-rich region. Upon binding to lipid-based membranes associated with microbes, this region exhibits significant changes in its secondary structure [35] (Figure 1, Table 1).

### 3.2. Antimicrobial and Antibiofilm Activity

Cationic AMPs act primarily as antimicrobial agents via direct interaction with the negatively charged components of bacterial membranes [44]. Specifically, they initially form an electrostatic bond with LPS (lipopolysaccharide) from Gram-negative bacteria or with lipoteichoic acids found in the peptidoglycans of Gram-positive bacteria [45]. Once accumulated onto the membrane surface, the structure of cationic AMPs gives them the ability to be inserted into the lipid bilayer due to their amphipathic nature [46]. As such, the peptides can induce structural instability into the membrane in three major ways. According to the barrel stave model, AMPs are inserted perpendicular to the plane of the bilayer and accumulate into oligomers spanning the bilayer [47]. The hydrophobic face of the peptide interacts with the lipid core of the bilayer, while the hydrophilic face lines up in an aqueous channel in the center of the bilayer. Therefore, for each molecule of peptide to be fully effective, it must have at least 22 amino acids, which would allow it to completely cross the bilayer [48]. A second model, the toroidal pore model, states that once inserted into the membrane, AMPs induce extreme curvature of the lipid bilayer [47,49]. Each side of the bilayer then bends continuously until it meets in the middle, forming a torus-shaped pore. The pores are formed and lined simultaneously by both the intercalated peptides and by the polar heads of phospholipids [50].

In addition to causing lysis of cellular membranes, some AMPs can enter the cytoplasm through the membrane and target highly specific molecules in the cell, where they function to halt macromolecular synthesis [51]. Certain types of AMPs (for example, indolicidin) bind specifically to either DNA and/or RNA, thereby preventing replication and transcription of genetic material [52]. Other AMPs selectively bind to ribosomes and stop translation and protein elongation. This action has been observed in proline-rich AMPs (PR-39, Bac5). Still other AMPs work to block the formation of peptidoglycan layers around cells by binding to lipid intermediates that are required for this process, rather than inhibiting enzymes involved in its production [53]. These include AMPs like tuberactinomycins. Biofilms, which are structured groups of cells encapsulated within a self-produced EPS matrix that provides resistance to most drugs used against bacteria, provide a unique challenge for drug treatment [54].

### 3.3. Immunomodulatory Functions

**Figure 2:**
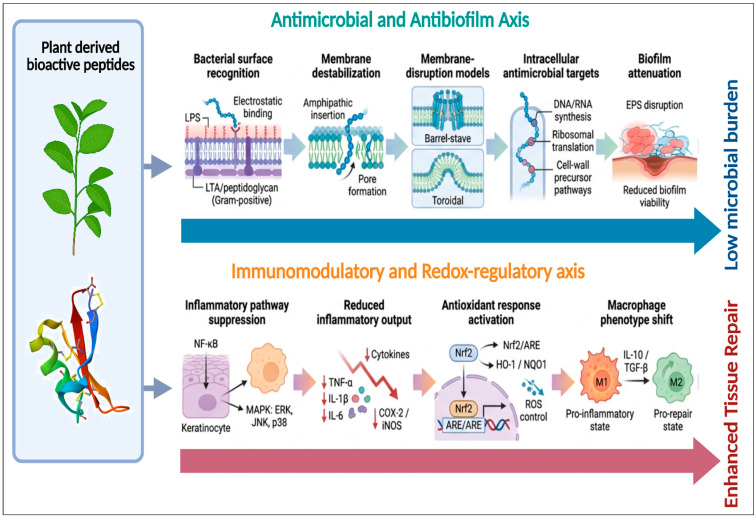
Antimicrobial and immunomodulatory mechanisms of plant-derived bioactive peptides in infected skin. The figure shows proposed mechanisms by which plant-derived peptide-based therapeutics may act in infected and inflamed skin. These include microbial membrane disruption, antibiofilm activity, modulation of NF-κB/MAPK signaling, activation of Nrf2/ARE antioxidant responses, cytokine regulation, and macrophage phenotype switching. Created in BioRender. Zaman, W. (2026). https://BioRender.com/9otxns6. accessed on 31 May 2026.

The use of cytokine modulation and control of inflammation through plant-derived bioactive peptides and polyphenol complexes is a mechanism through which plant-derived compounds can modulate immune function [55]. These mechanisms occur at the molecular level, where the compounds interact with intracellular signaling cascades and modulate cytokine expression [56]. Specifically, the compounds inhibit the activation of the major pro-inflammatory signaling cascades, i.e., NF-κB and MAPK; and the three major branches of the MAPK cascade, i.e., ERK, JNK, and p38 [57]. The inhibition of the activation of these pro-inflammatory signaling cascades leads to a decrease in the transcriptional activity of genes involved in the synthesis of pro-inflammatory cytokines such as TNF-α, IL-1β, and IL-6, and pro-inflammatory mediators such as COX-2 and iNOS [58]. The plant-derived compounds activate the Nrf2/ARE pathway, which increases the transcriptional activity of antioxidant and detoxification enzymes, e.g., heme oxygenase-1 (HO-1) and NAD(P)H quinone oxidoreductase 1 (NQO1), thereby restoring redox balance and reducing oxidative stress, the major driving force behind chronic inflammation [59]. Also, the interaction between plant-derived compounds and macrophages is another mechanism through which they influence immune responses [60]. They promote a shift from a pro-inflammatory (aerobic glycolytic) to a pro-repair (oxidative phosphorolytic) metabolic phenotype in macrophages [61]. With respect to this phenotypic shift, there is an increase in the secretion of anti-inflammatory cytokines such as IL-10 and TGF-β, leading to the resolution of chronic inflammation and the establishment of conditions that support repair processes [62] (Figure 2).

### 3.4. Limitations in Native Form

Although plant-derived peptides have considerable therapeutic utility, numerous physicochemical/biopharmaceutical limitations make it difficult to translate native peptides clinically [63]. One significant limitation of native peptides is that they undergo proteolytic cleavage. Native peptides are generally linear and readily degrade through enzymatic cleavage via the myriad of enzymes/proteases/peptidases found in skin/wound fluids [64]. In addition to being broken down (cleaved) rapidly, the half-lives of native peptides are also reduced significantly in an in vivo environment. Because of their high degree of water solubility, charge, and large size, native peptides experience low cutaneous permeability [65]. The “brick and mortar” structure of the stratum corneum is a highly organized and lipophilic structure, which is extremely restrictive in terms of the movement of hydrophilic substances and larger-sized peptides [66]. Thus, while many native peptides can potentially cross cell membranes, their inability to reside within the lipid-rich intercellular matrix of the skin results in little concentration within the viable epidermis/dermis [67]. Ultimately, all these limitations result in very poor cutaneous retention for native peptides. Since native peptides cannot easily embed themselves within or penetrate the superficial skin barrier without some form of additional assistance, they are quickly removed/degraded at the point of application [65]. Therefore, there exists a need for innovative structural modifications or “smart” delivery systems to protect the peptide from degradation, increase its ability to partition within cell membranes, and extend its retention time within the targeted tissue.

The translational value of plant-derived AMPs is uneven and should not be inferred from antimicrobial potency alone. Cyclotides, knottin-type peptides, and selected defensins have a stronger development rationale because cysteine-stabilized folds and cyclic or knotted architectures can improve structural stability, protease resistance, and sequence-engineering tolerance [23,68]. However, structural stability does not lessen the requirement for skin-relevant safety testing since the stable peptide scaffolds can still raise concerns related to immunogenicity, allergenicity and formulation complexity. Hevein-like peptides provide a focused antifungal rationale through chitin binding, but this mechanism may be less suitable for polymicrobial wounds dominated by bacterial biofilms [69]. Thionins and some puroindoline-derived peptides show strong membrane-disruptive activity, yet the same property creates a selectivity problem because excessive cationicity and hydrophobicity can increase host-cell membrane interaction, hemolysis, and mammalian-cell toxicity [70,71]. Snakins, lipid transfer proteins, α-hairpinins, and plant protease-inhibitor peptides remain useful discovery scaffolds; however, their cutaneous translation needs stronger evidence for their mechanism, host-cell selectivity, allergenicity risk and activity in wound-relevant biofilm models [72,73]. Therefore, the prioritization of “candidates” should integrate minimum inhibitory concentration, antibiofilm activity, protease stability, selectivity index, cytocompatibility, microbiota compatibility and formulation feasibility rather than relying on planktonic antimicrobial activity alone.

## 4. Engineering Plant-Based Peptide–Polymers

### 4.1. Polymerization and Conjugation Strategies

**Figure 3:**
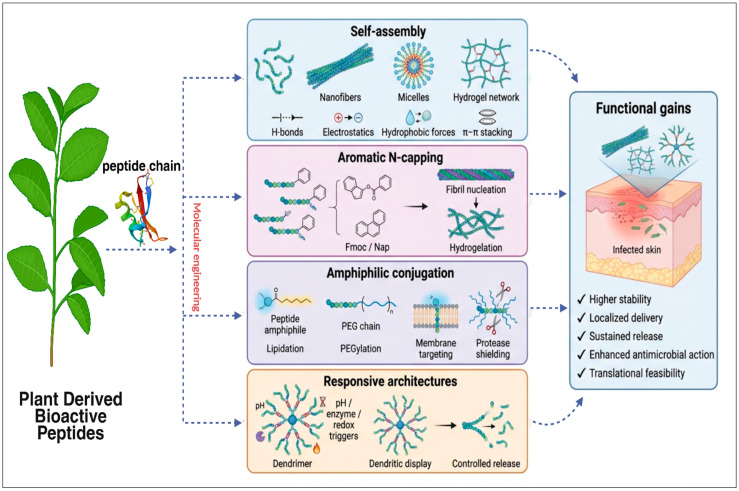
Engineering strategies for plant-based peptide–polymer therapeutics, including key molecular strategies used to improve peptide stability, local retention, and delivery performance. These include cyclization, D-amino acid substitution, PEGylation, lipidation, aromatic capping, dendrimerization, self-assembly, and polymer conjugation. Created in BioRender. Zaman, W. (2026). https://BioRender.com/9otxns6. accessed on 31 May 2026.

Peptide-based therapeutic development requires significant molecular design to address the inherent physical/chemical properties of natural peptides [74,75]. One very effective method of improving molecular organization involves the use of self-assembly to produce ordered structures such as nanofibers, nanotubes, nanospheres, and hydrogels [76]. Self-assembly is driven by thermodynamically favored non-covalent interactions such as hydrogen bonds, electrostatic forces, van der Waals forces, and π-π stacking [77]. In order to improve self-assembly potential, many peptides have been modified via N-capping with aromatic functional groups such as 9-fluorenylmethyloxycarbonyl (Fmoc) or naphthalene (Nap) [78]. As large, hydrophobic functional groups, they introduce an additional driving force for self-assembly based on strong π-π stacking and hydrophobic interactions [79]. These functional groups quickly nucleate stable 3D networks of fibrils that function as hydrogelators [80]. The peptide amphiphiles (PAs) include a hydrophilic peptide head and a hydrophobic lipid tail [81]. Therefore, they will self-assemble into cylindrical nanofibers or core-shell micelles while protecting the bioactive peptide sequence from degradation. Covalently attaching molecules to peptides has been shown to increase the resistance of the peptide to hostile physiological conditions [82]. Polyethylene glycol (PEG) attachment to peptides, known as PEGylation, increases both the size and the hydrodynamic radius of the peptide [83]. This results in an increased half-life for the peptide, provides protection from proteolysis, and reduces undesired immune responses. Lipidated peptides also provide greater localized concentrations of the peptide to target bacterial membranes and enhanced bacteriocidal activity. Stimuli-responsive covalent linkers enable the construction of multifunctional therapeutics that release active peptides when exposed to defined environmental signals [84]. Hyperbranched dendritic polymers represent a third dimension of structure and architecture that enables further manipulation of molecular organization [85]. Dendrimers are tree-like polymeric nanostructures (typically 2–5 nm) that contain a central core, multiple levels of branching macro-molecules, and large numbers of functional end-groups [86] (Figure 3).

### 4.2. Structure–Activity Relationships

The degree to which peptide–polymers interact with the pathogen target is determined by structural characteristics that reflect the balance of charge density, hydrophobicity and amphiphilicity [87]. Charge density is the primary driving force behind the interaction of the initial peptide-targeting event, which occurs between the positively charged peptide and negatively charged molecules found within the outer layer of bacterial cells [88]. These include lipopolysaccharides in Gram-negative bacteria and teichoic acids in Gram-positive bacteria. To facilitate this interaction, engineered peptides typically have a net positive charge (+2 to +9), most often created through incorporation of positively charged amino acids including cationic amino acids lysine and arginine [89]. Among these, arginine is particularly potent due to the ability of its guanidinium side chain to form two types of hydrogen bonding and π-cation interactions with lipid phosphate groups. As both the amount of positive charge and the proportion of hydrophobic residues increase in the sequence of the peptide, so too does the capacity of the peptide to inhibit bacterial growth [90]. There is a point of maximum positive charge where increasing amounts of positive charge will lead to a disproportionate increase in the likelihood of hemolytic tendencies and cellular toxicity against mammalian cells [91].

Hydrophobicity determines how readily the peptide can insert itself into and disrupt the nonpolar interior region of the microbe’s lipid bilayer [64]. Incorporating sufficient levels of hydrophobic residue (typically 30–50% of the sequence with residues such as tryptophan, phenylalanine, and leucine) into the peptide enables it to eventually become integrated into the lipid bilayer after first interacting via electrostatic forces [90]. Excessive hydrophobicity causes nonspecific binding, significant toxic effects on mammalian cells (hemolysis), and self-aggregation of the peptide in solution, thereby reducing antimicrobial availability [64]. Amphiphilicity, also referred to as the “hydrophobic moment,” describes how the arrangement of hydrophobic and hydrophilic amino acids creates a segregated distribution across the folded secondary structure of the peptide (for example, an alpha helix or beta sheet) [92]. When a linear polypeptide first comes into contact with the bacterial membrane, there is an immediate transition to a much more ordered secondary structure with clearly defined hydrophobic and hydrophilic faces [93].

### 4.3. Functional Optimization for Skin Delivery

To successfully transition plant-based peptide polypeptides into topical drug delivery systems, we have to overcome both of the harsh conditions found on and beneath the skin surface [94]. The first condition is the substantial barrier that exists in the form of physical properties of the skin; i.e., thickness, density, hydration, etc. Second, there is an abundance of proteases in wound exudates, which will quickly degrade any linear native peptide used as a therapeutic agent [95]. We can restrict proteolysis of the peptide by structuring it so that it cannot adopt the specific conformations that would allow for enzymatic cleavage. Cyclizing the backbone of the peptide with either head-to-tail or side chain-to-backbone linkages restricts conformational changes to the degree that they render the peptide resistant to proteolytic cleavage while maintaining its most bioactive conformation [96]. Incorporation of d-amino acids or other non-proteinogenic amino acids into the peptide sequence makes it impossible for mammalian and bacterial proteases to recognize these sequences [97]. Incorporation of n-methyl groups onto the peptide backbone adds steric bulk to inhibit access of enzymes to the active site and limits the number of conformations available to the peptide [98]. In addition, n-methylation reduces intramolecular hydrogen bonding between different parts of the peptide molecule and increases the time required for the peptide to undergo proteolytic cleavage [99].

For effective application of this technology, we need to maximize cutaneous retention in order to maintain a sufficient concentration of the peptide at the wound site [100]. Cutaneous retention can be improved by encapsulating or electrostatically binding the optimized peptides into large molecular weight biopolymeric matrices such as chitosan or hyaluronic acid gels. Once bound into one of these matrices, the peptides remain at the wound site for extended periods of time because of several factors [46,101]. First, the matrix acts as a bioadhesive, providing a strong bond to the wound surface. Second, when excess fluid accumulates at the wound site, the matrix absorbs much of this fluid, reducing its effect on skin irritation and inflammation [102,103]. Due to a combination of slow permeation through the matrix and controlled release mechanisms, the matrix maintains a high concentration of the peptides at the wound surface throughout the proliferative phases of wound healing, thus avoiding ‘burst release’ phenomena that typically cause local toxicities [104].

Molecular engineering should be evaluated as a trade-off rather than as an automatic improvement. Cyclization, D-amino acid substitution, and backbone modification can improve resistance to proteolysis, but these changes may also alter folding, solubility, immune recognition, and biological activity [105]. Polyethylene glycol conjugation can reduce enzymatic degradation and increase hydrodynamic size; however, their excessive shielding can lead to decreased interaction with membranes, thereby lowering antimicrobial potential. Similarly, the lipidation can improve membrane affinity and local retention, but this can also favor hemolysis, aggregation and nonspecific binding to mammalian cells [71]. Nevertheless, the aromatic capping and dendrimerization can support self-assembly and multivalent activity, but these modifications require strict control of aggregation behavior, release kinetics, and batch reproducibility [106]. A clinically useful engineering strategy should therefore show a net gain across potency, protease resistance, release behavior, cytocompatibility, and manufacturability within the same experimental system.

## 5. Delivery Systems for Cutaneous Applications

### 5.1. Topical and Transdermal Platforms

Traditional and advanced topical formats, in the form of hydrogel gels, cream formulations, and film formats, have been extensively modified to improve direct access to the dermal surface by delivering peptides [107]. Hydrogel-based systems consist of either naturally occurring or synthetically created three-dimensional cross-linked matrices that mimic an ECM and provide high hydration levels [108]. Due to their ability to promote absorption of excessive exudate, support gas transfer through permeability control, and release peptides through diffusion or enzymatic degradation of the polymer matrix, these systems make ideal wound-dressing applications. Semi-solid emulsion products include cream and ointment formulations that are formulated to provide specific rheological properties that will stabilize the distribution of water and lipids within the product while also controlling the incorporation of the active pharmaceutical ingredient [109]. This provides a method for enhancing penetration of amphiphilic peptides into the skin layer by facilitating stabilization of the peptide at the lipid/water interface.

Films are another type of topical platform that provides structural differences from other types of topical platforms [110]. Films, which provide a level of semi-occlusion from environmental insults as well as localized delivery of drugs at the point of contact, provide an opportunity for improving efficacy when used in conjunction with concentrated amounts of bioactive agents [110]. When formulated using biocompatible materials like PVA and chitosan, these thin, flexible films may be engineered to bind antimicrobial peptides on a molecular basis through either covalent bonding or electrostatic attraction [111]. By placing high concentrations of bioactive molecules at the film/tissue interface, films cause localized disruption of membranes on pathogenic microorganisms located in close proximity to the site of application while minimizing the loss of peptide activity throughout the remainder of the wound bed [112].

### 5.2. Nanocarrier-Based Systems

Peptide Therapeutic Dermal Bioavailability is primarily changed by the use of peptide therapeutic nanocarriers, which allow for a different drug exposure profile and a greater dermal bioavailability than traditional peptide therapies by avoiding the inherent limitations of the skin’s macroscopic barrier [113]. Peptides are commonly incorporated into polymeric nanoparticles made from biocompatible, degradable polymers such as chitosan, dextran, or poly (lactic-co-glycolic acid) (PLGA). These particles may be designed to enclose hydrophilic and amphiphilic peptides either inside the particle or on its surface, thus protecting peptides from enzymatic degradation by tissue proteolytic enzymes [114]. The ability of these nanoparticles to traverse the skin through appendageal routes (i.e., hair follicles) and through paracellular pathways due to their small size (submicron) and ability to have tunable surface charges also allows for a sustained release of peptides, allowing peptides to penetrate to deeper tissue layers and combat chronic infection or biofilm formation with less risk of causing significant host-cell cytotoxic effects [115,116].

**Figure 4:**
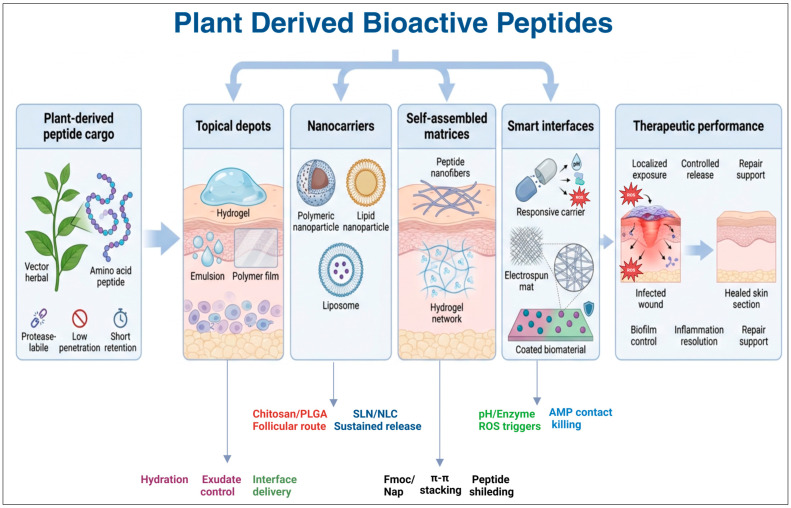
Cutaneous delivery platforms for plant-derived peptide–polymer therapeutics, including topical and transdermal platforms for improving peptide protection, skin retention, and controlled release. Representative systems include hydrogels, films, creams, liposomes, polymeric nanoparticles, electrospun fibers, self-assembling matrices, and surface-tethered biomaterials. Created in BioRender. Zaman, W. (2026). https://BioRender.com/9otxns6. accessed on 31 May 2026.

Lipid-based nanocarriers (solid lipid nanoparticles (SLNs), nanostructured lipid carriers (NLCs), and liposomes) are particularly well-suited for increasing the cutaneous delivery of amphipathic peptides [116]. SLNs and NLCs incorporate peptides into the lipid matrix of the stratum corneum, creating an occlusion that significantly increases the hydration level of the stratum corneum and causes a temporary disruption of the lipid lamella structure, thereby enhancing the ability of peptides to penetrate between cells [117]. Liposomes, having a phospholipid bilayer that is very similar in composition to that found in biological cell membranes, provide liposomes with several unique capabilities. Specifically, liposomes can encapsulate both hydrophilic peptides in the aqueous core and hydrophobic peptides in the phospholipid bilayer. This unique biomimetic design provides liposomes with a high degree of flexibility regarding how they interact with cellular membranes [118]. Thus, liposome fusion with microbially derived or mammally derived cell membranes is greatly enhanced, leading to targeted intracellular delivery of peptides, prevention of peptide degradation, and, ultimately, reduction in the required therapeutic dose [119] (Figure 4).

### 5.3. Self-Assembling and Stimuli-Responsive Systems

Self-assembling peptides are one of the most advanced classes of supramolecular biomaterials, which can be designed to self-organize into hierarchically structured nanoforms, for example, nanofibers, nanotubes, and micellar structures [120]. This spontaneous organization is typically driven by thermodynamically favored intermolecular forces such as hydrogen bonds, hydrophobic forces, and pi-pi stacking. Peptide sequences containing aromatic capping motifs (for example, Fmoc or naphthalene) or alternating patterns of hydrophilic and hydrophobic amino acids will assemble spontaneously in aqueous solutions, forming highly rigid hydrogel matrices capable of entraining substantial amounts of water [121]. The hierarchical structure formed has an intrinsic capacity to protect encapsulated drugs from rapid enzymatic degradation within the biological environment, concurrently providing a biocompatible depot for large doses of therapeutically active pharmaceutical agents [94].

Smart drug delivery systems further enhance this capability through their ability to achieve controlled spatial and temporal release of therapeutic agents based solely on physiological stimuli present in the pathological micro-environment [122]. Such smart drug delivery systems utilize enzyme-instructed self-assembly (EISA) technology, where enzymes that have been shown to be up-regulated at sites of infection or cancer (for example, phosphatase, esterase, MMP, etc.) catalytically drive the breakdown or conformational change in the peptide matrix to liberate the encapsulated payload [123]. Alternatively, other types of smart drug delivery systems may employ pH-responsive or temperature-responsive systems that take advantage of the acidification (pH 5–6) and oxidative stress associated with inflammation or neoplasia to initiate site-specific breakdown or transition of the peptide-based carrier system to provide high-dose local drug treatment while maintaining the structural integrity of adjacent healthy tissue [124].

### 5.4. Surface-Tethered and Biomaterial-Integrated Systems

Antimicrobial peptides have been integrated into larger-scale biomaterial scaffolding to transition their use from passive containers to active interfaces [125]. Utilizing electrospun polymers such as PVA and/or cellulose acetate, peptide delivery matrices in the form of extremely thin nanofiber mat have achieved the highest possible surface area-to-volume ratios, which simultaneously allow for effective management of exudates and control of drug-released kinetics [126]. The goal of each specific application will determine if the peptides are used through physical adsorption as an immediate “burst” release mechanism to address acute microbial load or covalent attachment via orthogonal spacers (i.e., Maleimide Linker) to provide sustained, contact-based eradication of pathogens while concurrently promoting hemostatic and angiogenic effects [127].

The primary applications of surface-bound peptide systems for implantable medical devices involve engineering these systems to prevent infection and biofilm development associated with the implanted device [128]. Antimicrobial peptides are chemically bound to various types of material surfaces, including titanium, silicones, and stainless steel, utilizing anchoring techniques such as silane modification, click chemistry, or grafting of polymer brushes [129]. These systems generate durable, non-adherent, contact-killing functional coatings that inhibit microbial adherence and subsequent colonization without changing either the bulk mechanical or biological compatibility characteristics of the original device [130]. It is essential that appropriate densities of immobilized peptides, along with optimized length of spacers between the peptide and substrate, maintain sufficient mobility of the peptide domain to permit lysis of incoming microorganisms.

### 5.5. Skin Barrier Interaction

The stratum corneum is a formidable physical barrier that necessitates a sophisticated biophysical approach to allow for the penetration of macromolecular therapeutic peptides [67,131]. The skin acts as a barrier limiting the passage of transdermally administered drugs through its ‘brick and mortar’ structural arrangement of keratinized corneocytes and lipid lamellae [113,132]. However, transdermal peptides can selectively penetrate the skin barrier using specific targeted amino acid sequences rich in cationic (arginine and lysine) and hydrophobic (tryptophan and phenylalanine) residues to selectively disrupt the highly ordered lipid matrix on a temporary, reversible basis [133]. In terms of mechanism of action, SKPs create localized nano-permeable channels within the lipid matrix of the SC through both: (i) electrostatic adsorption/interfacial anchoring of peptides at the interface of lipids, leading to a reduction in the phase transition temperature of lipid molecules, thus creating transient nanoscale permeation channels; and/or (ii) targeting of trans-appendageal (hair follicle) routes to avoid interaction with the skin barrier altogether [134].

Innovative vesicle-based systems such as transferosomes and ethosomes interact with the skin barrier by emphasizing extreme deformability and lipid fluidization rather than relying solely upon passive diffusion across the skin barrier [135]. Transferosomes use edge activators to destabilize the lipid bilayer structure of the vesicles, enabling them to be squeezed through narrow intercellular pathways under transdermal hydration gradient forces. Ethosomes exploit high concentrations of ethanol to increase lipid fluidity of the *stratum corneum* [135]. These combined approaches enhance permeation and coordinate with keratin structures to provide sufficient amounts of peptide therapeutics into viable epidermis and superficial dermis without inducing long-term erosive damage to the skin [136].

Topical matrices, nanocarriers, electrospun fibers, vesicular systems, and surface-tethered biomaterials offer rational approaches to improve peptide delivery, yet their translational value depends on more than improved encapsulation or release. Clinically useful systems must preserve peptide integrity, maintain local concentrations within the therapeutic window, avoid burst-related cytotoxicity, and remain manufacturable under reproducible conditions. Many delivery studies still rely on simplified in vitro models that do not reproduce protease-rich wound exudate, polymicrobial biofilms, variable pH, oxidative stress, and impaired perfusion. Therefore, delivery systems should be evaluated using disease-relevant skin and wound models before therapeutic conclusions are drawn.

### 5.6. Comparative Translational Relevance of Delivery Platforms

The translational relevance of cutaneous peptide-delivery platforms differs according to clinical familiarity, manufacturing control, release behavior, and evidence quality. Hydrogels currently provide the strongest near-term rationale for infected and inflamed wounds because they can combine moisture balance, exudate absorption, local retention, and controlled peptide release within a wound-dressing format [101]. Their main limitations are peptide-loading reproducibility, sterilization, mechanical stability, burst-release control, and maintenance of peptide activity in protease-rich exudate. Liposomes, solid lipid nanoparticles, and nanostructured lipid carriers can improve peptide protection and controlled release, but translation depends on colloidal stability, encapsulation efficiency, storage stability, scalable production and defined quality parameters [137]. Polymeric nanoparticles offer controlled release and protease protection, but they add quality-control demands related to particle size, polydispersity, surface charge, residual solvent, peptide loading, and batch comparability [138]. Electrospun fibers provide high surface area and extracellular matrix-like architecture, yet scale-up, solvent removal, sterilization, fiber uniformity, and mechanical integrity remain major barriers [139]. Self-assembling peptide matrices and stimuli-responsive systems are mechanistically attractive because they can combine bioactivity with supramolecular organization, but their performance may be sensitive to pH, ionic strength, peptide purity, aggregation state, and wound-exudate composition [140]. Surface-tethered systems may be appropriate for contact-killing coatings, but they are less suitable when peptide diffusion into heterogeneous wound tissue is required [129]. Thus, hydrogels and established lipid or polymeric nanocarriers appear closer to near-term cutaneous translation, whereas electrospun, self-assembling, stimuli-responsive, and tethered systems require stronger manufacturing and disease-relevant validation before clinical claims are justified (Table 2).

## 6. Mechanistic Basis in Cutaneous Infections and Inflammation

In this section, mechanisms directly reported for plant-derived AMPs are distinguished from mechanisms inferred from the broader antimicrobial peptide and peptide–polymer literature. Where plant-specific evidence is limited, the discussion is presented as mechanistic extrapolation rather than direct evidence for plant-derived peptide systems.

### 6.1. Antibacterial and Antibiofilm Mechanisms

The first line of defense for cationic AMPs and plant-derived bioactive polymers is the electrostatic interaction between the peptides and negatively charged components of microbial membranes (lipopolysaccharides in Gram-negative bacteria and lipoteichoic acids in Gram-positive bacteria) [145]. Once the peptides accumulate at the membrane surface, their ability to insert themselves into the lipid bilayer due to the amphipathic nature of the peptides results in significant changes to the structure and stability of the membrane. The three most common models of how this occurs are the barrel stave model (formation of oligomeric channels across the membrane), the toroidal pore model (severe local curvature that merges adjacent leaflets of the bilayer), and the carpet model (catastrophic fragmentation of the bilayer into micelles) [146]. Along with disrupting the structural integrity of bacterial membranes, some peptides can also cross the cytoplasmic membrane and inhibit critical cellular functions, including protein synthesis, DNA replication, and the formation of important cell wall precursors. Biofilm-producing bacteria commonly found in chronic skin infections form complex structures consisting of multiple layers of attached bacteria embedded in a protective matrix produced from self-secreted polysaccharide (polymers of glucose, galactose, etc.) called extracellular polymeric substances (EPSs) [147]. These structures have significantly increased resistance to both host immune responses and to conventional antibiotic treatments. Synthetic peptide-based therapies utilize a multi-modal approach to eliminate these recalcitrant bacterial populations. First, engineered peptides break down structural elements of the EPS matrix, making it more porous, allowing other therapeutic agents to enter bacterial cells [148]. Second, the same engineered peptides induce rapid depolarization of bacterial membranes, resulting in rapid depletion of ATP stores, leading to “bioenergetic collapse” in stationary phase/sessile bacteria [112].

### 6.2. Immunomodulatory Pathways

Plant-based bioactive compounds and synthetic peptide–polymers have substantial immunomodulatory activity due to the interaction with pattern recognition receptors (PRRs) and significant modification of intracellular signal transduction [149]. A primary function in this regard is the suppression of pro-inflammatory pathways, particularly those associated with the activation of NF-κB and MAPK cascades (p38, JNK, ERK). The down-regulation of excessive production of pro-inflammatory mediators, including COX-2, iNOS, TNF-α, IL-1β, and IL-6, is also achieved by the targeted inhibition of pro-inflammatory transcription [150].

Oxidative stress is another central regulator of chronic wound inflammation. Excessive reactive oxygen and nitrogen species damage keratinocytes, fibroblasts, endothelial cells, and extracellular matrix components [151]. The Nrf2/ARE pathway counterbalances this injury by inducing antioxidant and cytoprotective enzymes, including heme oxygenase-1 (HO-1) and NQO1. Nrf2 activation can also limit inflammatory amplification by interacting with NF-κB-dependent signaling [152]. Thus, a therapeutically useful peptide–polymer system should ideally reduce excessive oxidative stress without suppressing the early antimicrobial immune response required for pathogen clearance.

Reversal of chronic skin inflammation requires the modulation of the innate immune system and re-programming of macrophages [153]. The aforementioned bio-instructive therapeutics are capable of reprogramming macrophages from M1 (pro-inflammatory) to M2 (pro-reparative) states. As macrophages undergo this transition, they alter their metabolism and cytokine profiles, leading to an increase in local production of anti-inflammatory cytokines (e.g., IL-10, TGF-β) responsible for the resolution of ongoing inflammatory processes and establishing a localized immune tolerance [62]. Persistent M1-like macrophage activity maintains high levels of inflammatory cytokines, oxidative stress, and protease activity, thereby delaying closure. In contrast, reparative macrophage states are associated with increased IL-10, TGF-β, arginase activity, and oxidative metabolism, which support inflammation resolution and matrix remodeling [154,155].

### 6.3. Wound Healing and Tissue Regeneration

Plant-based peptide–polymers can provide a way to promote the proliferative and remodeling phases of wound healing by stimulating key aspects of cellular behavior in the healing process [156]. Because of their action on cellular behavior, plant-based peptide–polymers can cause an increase in the number of cells that are present within the wound bed. More specifically, they can stimulate the proliferation and migration of dermal fibroblasts and epidermal keratinocytes, both of which are critical to the healing process [157]. Plant-based peptide–polymers can also increase the production of pro-angiogenic growth factors, most importantly VEGF, PDGF, and bFGF. This molecular stimulation will lead to significant increases in the formation of new blood vessels, or angiogenesis [158]. Angiogenesis provides the oxygen and nutrients necessary for the survival and development of newly formed granulation tissue. In addition to influencing cell behavior and promoting angiogenesis, plant-based peptide–polymers can also directly influence how cells interact with each other and the surrounding environment. Extracellular matrix (ECM) remodeling is an important component of this interaction and is directly related to the restoration of anatomy and function to damaged skin [159]. Plant-based peptide–polymers can activate pathways that control how the ECM is remodeled. These pathways include TGF-β1/Smad and PI3K/AKT. Activation of these pathways leads to increased rates of de novo collagen synthesis by fibroblasts, along with proper alignment and crosslinking of collagen type I and III fibers [160]. Proper alignment and crosslinking result in greater tensile strength and elasticity of the healing tissue.

### 6.4. Skin Microbiota as a Therapeutic Determinant

Skin microbiota is directly involved in cutaneous inflammation, infection persistence and wound repair. Healthy skin is commonly colonized by commensal bacteria such as *Staphylococcus epidermidis*, *Staphylococcus hominis*, *Cutibacterium acnes*, *Corynebacterium* spp., *Micrococcus* spp., as well as fungi such as *Malassezia* spp. [161]. The relative abundance of these common microbes generally varies according to skin site, hydration, sebum content, pH, age and disease states [162]. In healthy skin, these commensals contribute greatly towards barrier homeostasis, colonization resistance and immune calibration, whereas dysbiosis can amplify inflammatory signaling and promote pathogen overgrowth. On the other hand, in chronic wounds, the microbial imbalance and polymicrobial biofilms are mainly related to delayed healing, persistent inflammation and reduced antimicrobial responsiveness [163,164]. Antimicrobial peptides also interact with skin microbiota by restricting pathogen growth, thereby facilitating balance of commensal microbes [165]. Therefore, plant-derived peptide–polymer therapeutics should be evaluated not only for broad antimicrobial activity but also for their ability to suppress pathogenic biofilms.

### 6.5. Diet and Nutritional Status as Systemic Modifiers of Cutaneous Repair

Diet and nutritional status may influence cutaneous inflammation, infection susceptibility and wound repair [166]. This is probably due to modulation of systemic immunity, oxidative stress, collagen synthesis, angiogenesis, glycemic control and host–microbiome interactions. An adequate protein intake and micronutrients (vitamins A, C, D, and E, zinc, selenium, and iron) are relevant and are considered important for immune competence, extracellular matrix formation and tissue regeneration, whereas malnutrition can delay healing [167]. In cases of diabetic foot ulcers and chronic wounds, a detailed assessment of nutritional status is of prime concern since protein deficiency and inadequate micronutrient status can lead to impairment of inflammation and tissue repair [168]. However, current evidence does not directly establish that diet modifies the efficacy of plant-derived peptide–polymer therapeutics. Therefore, diet should be considered a systemic host factor.

The mechanistic evidence for plant-derived peptide–polymer systems remains fragmented. Antimicrobial, antibiofilm, immunomodulatory, antioxidant and wound-repair effects are often reported separately. This makes it hard to interpret whether the major therapeutic outcomes are relevant to improved tissue repair, microbial killing, and biofilm disruption. This distinction is important since an excessive membrane disruption can on one side improve antibacterial activity and also reduce host-cell compatibility on the other hand. Future mechanistic studies should combine microbial assays, host-cell inflammatory markers, biofilm models, and wound-healing endpoints in the same experimental design to establish causal links between material properties and biological outcomes.

## 7. Translational Challenges (Critical Analysis)

### 7.1. Stability and Degradation in Skin Microenvironment

Native antimicrobial peptide molecules and botanical extract-based materials undergo very high degrees of physical instability and biological instability within the hostile environment of the skin and wound [169]. A major barrier to translating native AMPs and botanically derived therapeutic agents to the clinic is that they have a rapid rate of proteolytic degradation due to the presence of endogenous serine protease enzymes (e.g., KLK5 and KLK7), as well as matrix metalloproteinases (MMPs) in both the epidermis and wound exudate fluids [170]. These enzymatic activities lead to the rapid cleavage of native AMPs into inactive fragments; therefore, their in vivo half-lives are extremely short. Moreover, the inherent low water-solubility properties of many plant-derived bioactive compounds such as polyphenols and carotenoids make them subject to significant rates of thermal degradation, photo-oxidation and pH-dependent chemical changes [171]. There is considerable uncertainty regarding how much of these compounds will remain active after topical application to the skin. Without employing either structural modification techniques or encapsulating these large-molecule bioactives within a biocompatible advanced delivery system, it has been demonstrated that they fail to penetrate through the stratum corneum barrier and are eliminated from the body before achieving therapeutically relevant levels of concentration at the target site(s) [172].

### 7.2. Toxicity, Irritation, and Selectivity

The use of natural polymer peptides in clinical settings has been largely hindered due to several problems, including cytotoxic effects on the cells hosting them, as well as irritation locally, and a lack of specificity [173]. As they are highly cationic and have an amphipathic nature, some peptides are capable of effectively breaking through microbial membrane barriers but can cause both dose-related hemolytic reactions and significant cytotoxic effects by interacting randomly with mammalian cell membrane barriers [174]. Also, applying the numerous and complex mixtures of various botanical extracts has its own dangers of eliciting an allergic reaction upon contact (contact dermatitis), as well as phototoxicity and photoallergenic reactions, especially if high concentrations are used or subjected to ultraviolet light. In addition, there exists a large risk that will be caused by the unintended overactivation of the immune system [175]. For example, contaminations with endotoxins from plants used in developing biomaterials can activate the TLR4 receptors from false signals, whereas excessive concentrations of peptides can elicit chronic inflammatory signaling and inhibit tissue repair.

For chronic wound applications, safety assessment should extend beyond single-dose cytotoxicity. Peptide–polymer conjugation can increase tissue residence and protease resistance, but repeated exposure on barrier-impaired skin may increase immune recognition, complement activation, cytokine release, delayed hypersensitivity, or irritant dermatitis [112]. Broad-spectrum membrane-active peptides may also disturb commensal skin microbiota and reduce colonization resistance [176]. Although antimicrobial peptides are generally considered less prone to resistance than conventional antibiotics, repeated sublethal exposure can still select for membrane-charge modification, protease production, efflux responses, biofilm adaptation, or cross-resistance to other membrane-active agents [177]. Therefore, chronic-wound translation should include repeated-dose dermal toxicity, sensitization testing, cytokine/complement profiling, microbiome analysis, serial-passage resistance assays, and antibiofilm recurrence models.

Safety assessment should be framed around exposure pattern, peptide mechanism, and wound condition rather than single-dose cytotoxicity alone. Membrane-active peptides can reduce microbial viability, but the same physicochemical features that support bacterial membrane disruption, especially high cationicity and hydrophobicity, can increase host-cell membrane interaction, hemolysis, keratinocyte injury, fibroblast toxicity, irritation, or delayed re-epithelialization [71,178]. Polymer conjugation and nanocarrier encapsulation may reduce proteolysis and prolong tissue residence, but increased risks of cytokine release sensitization and persistent inflammation can occur upon longer exposure. Antimicrobial peptides interact with the skin microbiota and this interaction can influence commensal balance as well as host defense [165]. Therefore, cutaneous translation should include comprehensive safety and toxicity assessments.

### 7.3. Manufacturing and Scalability Constraints

Transitioning plant-based peptide therapeutic compounds from bench scale to commercial production is riddled with major manufacturing and scaling challenges [63]. Peptide synthesis in a laboratory setting, using mainly solid phase peptide synthesis (SPPS) techniques, can be very costly and time-consuming because of the expensive starting material used in these methods, the need for rigorous purification procedures, and the large amounts of hazardous organic chemicals that are utilized during large-scale production [179]. On the other hand, extracting peptides and other bioactive molecules directly from plants is plagued with inherent low yield levels, numerous complex steps required to isolate these compounds, and tremendous differences in the quality of extracts produced from harvests grown on different continents, at various times of the year, and under different growing conditions [180]. In addition, creating advanced composite nanoparticle delivery systems in compliance with good manufacturing practices (GMPs) has scaling issues since controlling both the exact size of nanoparticles, the amount of peptide loaded into each nanoparticle, and the structure of each matrix composition is difficult and extremely expensive for large-scale commercialization [181].

### 7.4. Limited In Vivo and Clinical Evidence

The major hurdle in translating plant-peptide therapeutics to the human scale for clinical use has been the severe lack of numerous large-scale randomized human clinical trials establishing both the efficacy and safety of these therapies [182]. The majority of evidence used today to support this therapeutic class is generated through in vitro assays (reductionist) and pre-clinical animal model-based research [183]. These types of studies poorly replicate the significant spatial heterogeneities, metabolic gradients, and specialized immune environments present within the human skin and chronic wound tissues [184]. It is well known that results achieved in preclinical models rarely correlate with positive human outcomes due to variations in cutaneous structure, limited bioavailability of peptides at human wound sites, as well as the highly developed evasion mechanisms by polymicrobial biofilm populations [185]. In addition, all of the few clinical trials performed thus far have typically included very low numbers of subjects, limited treatment duration, variable composition of formulations, and inadequate standardization of clinical endpoints, therefore limiting comparison across studies and providing little opportunity to establish credible, evidence-based guidelines for therapy.

Currently, the most clinically informative peptide examples come from non-plant antimicrobial peptide systems. Topical pexiganan cream was evaluated in randomized, double-blind trials for mildly infected diabetic foot ulcers and showed clinical outcomes comparable to oral ofloxacin in combined analyses [186]. In contrast, topical omiganan was safe and well tolerated in a randomized study of facial seborrheic dermatitis but did not produce clear clinical improvement compared with placebo [187]. These examples show that topical peptide delivery is clinically feasible, but they also illustrate why human validation is necessary before broad translational claims are made (Table 3).

### 7.5. Regulatory and Cost Considerations

Commercialization of botanical peptide–polymer therapeutic products is impeded by very strict, and many times fragmented, worldwide regulatory systems [194]. Much of this complexity comes from a lack of clear regulatory classification for the novel hybrid material of peptide–polymers, as they can be classified as either cosmetic products or medical devices, depending upon what claim they are made to address and how they work [17]. Each agency that regulates them has required documentation regarding safety, efficacy, and batch-to-batch consistency. Because botanical extract-derived materials have such high levels of heterogeneity in terms of composition, the process of obtaining the level of documentation needed to obtain regulatory approval (e.g., the necessity for broad-based toxicology studies, long-term clinical trials, and Good Manufacturing Practices) increases both time and cost [58]. These significant increases in both time and cost make it extremely difficult for Small and Medium-Sized Enterprises (SMEs), etc., to move new therapeutics to clinical use [195,196] (Table 4).

Regulatory classification of plant-derived PMPs depends on intended use, primary mode of action, and jurisdiction. In the FDA framework, these products may fall under drug, device, biologic, or combination-product pathways depending on whether the main effect is pharmacological, biological, or physical. FDA defines combination products as products composed of drug, device, and/or biological components, with classification guided by primary mode of action. In the EMA/EU framework, medicinal products used with medical devices require quality documentation for the device component when it may affect product quality, safety, or efficacy [197,198]. Nanocarrier-containing peptide systems require defined critical quality attributes, including particle size, surface charge, peptide loading, release kinetics, sterility, residual solvents, and storage stability, because nanomaterial-containing products can be highly sensitive to process conditions and scale [199]. Botanical-derived materials further require control of plant identity, source, extraction, peptide fingerprinting, impurity profile, and batch comparability.

### 7.6. Commercialization and Clinical-Translation Readiness

The clinical translation of plant-derived peptide–polymer therapeutics depends on more than antimicrobial potency. Most clinically advanced antimicrobial peptides are microbial, synthetic, or animal-derived rather than plant-derived; therefore, their success cannot be directly extrapolated to plant peptide systems [200]. Approved or clinically investigated peptide antimicrobials show that peptide-based anti-infectives can reach clinical use, but they also highlight persistent barriers, including proteolytic instability, cytotoxicity, weak in vivo efficacy and complexities in formulation design [193,201].

Among various key challenges, reproducible sourcing is of prime concern in plant-derived peptide science. This is because disulphide connectivities, overall yield, purity and folding of plant peptides vary with genotypes, cultivation modes, harvesting stages and extraction methods [202]. Clinical development therefore requires validated identity testing, impurity profiling, potency assays, and batch-to-batch comparability. Solid-phase synthesis, recombinant expression, and molecular farming may improve reproducibility, but each approach introduces cost, scale-up, folding, purification, and regulatory constraints [203].

Furthermore, the reproducibility of the formulation is equally important. Investigations have shown that for peptide delivery, the design and choice of polymer and incorporation modes are critical and these are based on peptide loading, particle size or network structure, release kinetics, sterility and cytocompatibility, etc. [204]. Regulatory classification may also be complex because these products can combine pharmacological peptide activity with device-like functions such as wound coverage, exudate control, or controlled release. Intellectual-property protection and economic feasibility remain additional barriers, particularly when native plant peptide sequences offer limited patentability [193]. Thus, a finished peptide-based therapeutic agent must possess antimicrobial activity, dermal safety, formulation stability, reproducibility and clear regulatory positioning.

**Table 4:** A detailed overview of validation models and translational challenges for plant-derived peptide–polymer therapeutics in cutaneous infection and inflammation.

Translational Barrier	Main Cause	Key Risk	Design Response	Validation Approach	Ref.
Proteolytic degradation	KLK-mediated AMP cleavage	Loss of activity	D-amino acids; cyclization; capping	LC–MS; residual MIC	[205]
Wound-environment instability	Hydrolysis, oxidation, photolysis, alkaline pH	Short topical activity	Hydrogel/nanocarrier protection	Stability + release assay	[206]
Poor skin retention	Low residence time; barrier effects	Subtherapeutic exposure	Bioadhesive films; hydrogels	Franz diffusion; retention assay	[207]
Weak selectivity	Charge/hydrophobicity imbalance	Hemolysis; cytotoxicity	Tune charge, amphipathicity, PEGylation	HC_50_; IC_50_; SI	[71]
Botanical sensitization	Plant allergens/photosensitizers	Contact dermatitis	Purified fractions; allergen profiling	Patch/photopatch test	[208]
Immunotoxic contamination	LPS/bioburden in formulations	False inflammation signal	Endotoxin-free GMP processing	LAL; TLR4; cytokines	[209]
Biofilm tolerance	EPS barrier; redox stress	Persistent infection	Antibiofilm hydrogel matrix	MBEC; CLSM biofilm assay	[210]
Peptide synthesis burden	SPPS solvent/reagent intensity	High cost; low scalability	Greener SPPS; shorter analogues	Yield; purity; E-factor	[211]
Plant extract variability	Genotype, season, extraction shifts	Batch inconsistency	HPLC/LC–MS fingerprinting	Marker assay; chemometrics	[212]
Nanocarrier scale-up	Size, PDI, loading variability	GMP failure	QbD; defined CQAs	PDI; zeta; loading; sterility	[213]
Model mismatch	Rodent wounds differ from humans	Poor prediction	Porcine/ex vivo human models	Human-relevant endpoints	[214]
Limited clinical evidence	Few clinically advanced AMPs	Uncertain efficacy	RCTs; standardized endpoints	Healing, bacterial load, safety	[201]

## 8. Emerging Design Strategies and Future Directions

**Figure 5:**
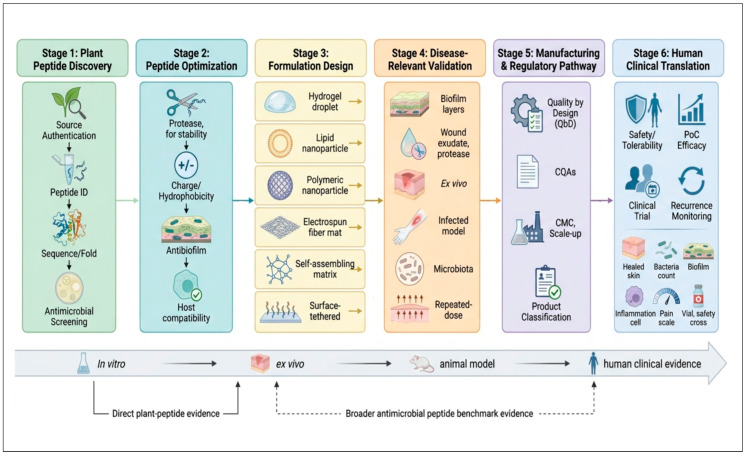
Translational roadmap for plant-derived peptide–polymer therapeutics in cutaneous infection and inflammation. The roadmap summarizes the stepwise progression from plant peptide discovery and molecular optimization to formulation design, disease-relevant preclinical validation, manufacturing control, regulatory assessment, and clinical evaluation. Created in BioRender. Zaman, W. (2026). https://BioRender.com/9otxns6. accessed on 31 May 2026.

AI-assisted peptide discovery, smart biomaterials, wearable patches, and microneedle-based delivery systems offer important opportunities for future development of plant-derived peptide–polymer therapeutics [215]. However, these technologies should be viewed as early-stage enabling tools rather than clinically validated solutions. AI and machine-learning models can accelerate peptide screening and help predict activity, stability, and toxicity, but their outputs remain limited by dataset bias, inconsistent training data, limited interpretability, and the need for experimental confirmation [216]. Similarly, microneedles, stimuli-responsive polymers, and sensor-integrated patches may improve local delivery and wound monitoring, but their translation requires reliable peptide loading, controlled release, mechanical robustness, sterilization, sensor accuracy, scalable manufacturing, regulatory clarity, and clinical validation [217,218]. Future progress should therefore follow a staged translational pathway that links computational prediction, peptide engineering, formulation reproducibility, disease-relevant preclinical models, dermal safety testing, and controlled clinical evaluation (Figure 5). This cautious framework better reflects the current maturity of these technologies in dermatological peptide therapeutics.

These technologies should be interpreted according to the maturity level. AI/ML-assisted peptide screening, microneedle-mediated skin delivery and wearable wound biosensors are already under experimental validation; however, they are not clinically established for plant-derived PMP therapeutics [219]. AI-guided patient-specific peptide selection, sensor-integrated peptide release and stimuli-responsive microneedle platforms remain early-stage concepts and require comprehensive validation [217]. Fully autonomous closed-loop wound platforms can be considered as long-term possibilities rather than current clinical solutions.

## 9. Conclusions

Plant-derived antimicrobial peptides and their peptide–polymer analogues represent a promising therapeutic platform for cutaneous infections, chronic inflammation, and biofilm-associated wound pathology. Their intrinsic antimicrobial, antibiofilm, immunomodulatory, and tissue-repair potential is particularly relevant in the context of increasing antimicrobial resistance and impaired wound healing. However, native plant-derived peptides remain constrained by rapid proteolytic degradation, limited stratum corneum penetration, short cutaneous residence time, possible mammalian-cell toxicity, and insufficient formulation stability. Polymer-based engineering strategies, including hydrogels, lipid nanoparticles, electrospun scaffolds, stimuli-responsive matrices, and surface-tethered biomaterials, provide rational approaches to improve peptide stability, local retention, controlled release, and site-specific activity. These platforms may also support multimodal therapeutic effects by combining direct microbial membrane disruption, biofilm attenuation, inflammatory pathway modulation, redox regulation, and repair-associated macrophage responses. Nevertheless, translation remains limited by manufacturing cost, scale-up complexity, batch-to-batch variability of plant-derived materials, regulatory classification uncertainty, and the scarcity of well-designed human clinical studies. AI-assisted peptide discovery, QSAR modeling, machine learning-based toxicity prediction, and quality-by-design manufacturing can accelerate the identification of stable, selective, and scalable peptide candidates. Future development should proceed through a stepwise translational framework linking peptide selection, polymer design and biological validation. Candidate peptides should be prioritized according to antimicrobial selectivity, antibiofilm activity, protease resistance, cytocompatibility and compatibility with commensal skin microbiota. Delivery platforms should also be optimized using defined critical quality attributes. These formulations should be evaluated in clinically relevant infected-wound models before progression to repeated-dose dermal safety studies and controlled clinical trials. This mechanism-guided and quality-controlled strategy can be important for advancing plant-derived peptide–polymer platforms from promising biomaterial concepts toward clinically credible dermatological applications.

## Figures and Tables

**Table 1:** Classes and major sources of plant-derived AMPs with potential relevance for dermatological and wound-care applications.

Peptide Class	Main Botanical Sources	Key Features	Skin Applications	Ref.
Defensins	Wheat, barley, spinach, radish, pea	~45–54 aa; ~5 kDa; cationic; cysteine-stabilized αβ fold; four disulfide bridges	Antifungal and antibacterial scaffolds; potential relevance for infected wounds and microbiome-associated skin disorders	[23,24]
Cyclotides	Violaceae, Rubiaceae; *Oldenlandia affinis*, Viola spp.	28–37 aa; head-to-tail cyclic backbone; cyclic cystine knot; high thermal and enzymatic stability	Stable peptide scaffolds for topical peptide engineering and sustained delivery	[32]
Thionins	Wheat endosperm, barley, Nicotiana attenuata, black cumin	~45–47 aa; highly basic and hydrophobic; three to four disulfide bridges	Rapid membrane-disruptive antimicrobial action; possible topical anti-infective scaffold, but toxicity must be assessed	[36,37]
Hevein-like peptides	*Hevea brasiliensis*, Wasabia japonica, cereals	~4–5 kDa; cysteine-rich; conserved chitin-binding motif	Strong antifungal rationale through fungal cell-wall chitin targeting	[22,23]
Snakins	Potato and other Solanaceae	Cysteine-rich; 12 conserved cysteines; six disulfide bridges	Antibacterial and antifungal potential; mechanism still incompletely resolved	[38]
Non-specific lipid transfer proteins	Spinach, onion seeds, wheat, barley, tomato and other land plants	~6.5–10.5 kDa; α-helical; eight-cysteine motif; four disulfide bridges; lipid-binding cavity	Potential membrane-interacting defense proteins; skin translation requires allergenicity assessment	[39]
Puroindolines/puroindoline-derived peptides	Wheat and related cereals	Wheat endosperm proteins with tryptophan-rich lipid-binding domains; antimicrobial activity mainly linked to the Trp-rich region	Potential membrane-active antimicrobial templates; formulation and mammalian-cell selectivity require evaluation	[32,40]
Knottin-type peptides	Diverse angiosperms, including Cucurbitaceae, Rubiaceae, and Solanaceae	Small cysteine-rich peptides with inhibitor cystine-knot fold; high thermal and proteolytic stability	Stable antimicrobial scaffolds for peptide engineering and topical delivery concepts	[23,41]
α-Hairpinins	Cereals and diverse angiosperms	Short cysteine-rich peptides; helix–loop–helix fold; usually two disulfide bridges	Antimicrobial scaffolds with potential for selective topical anti-infective engineering	[42]
Plant protease-inhibitor peptides	Legumes, cereals, Solanaceae, medicinal plants	Peptide/protein inhibitors of serine, cysteine, or aspartic proteases; often cysteine-stabilized	May reduce pathogen virulence and protease-driven wound inflammation; useful as adjacent wound-care bioactives	[43]

**Table 2:** Evidence-based translational-readiness profile of delivery platforms for plant-derived peptide–polymer therapeutics.

Platform	Evidence Basis	Readiness Level	Main Limitation	Ref.
Hydrogels/wound dressings	Established wound-dressing device precedent	Strong platform readiness	Peptide loading, sterilization, burst release, antimicrobial claims	[141]
Liposomes/lipid nanocarriers	Defined CMC guidance for liposome drug products	Moderate–strong platform readiness	Particle size, encapsulation, lipid stability, release testing	[142]
Polymeric nanoparticles	Tunable drug-delivery platform with broad translational literature	Moderate readiness	Polymer identity, residual solvent, batch reproducibility	[138]
Self-assembling peptide matrices	RADA16/PuraStat clinical-use precedent as a hemostatic peptide hydrogel	Moderate platform readiness	pH/ionic sensitivity, aggregation, release control	[143]
Electrospun fibers	Strong preclinical wound-dressing evidence	Moderate–low readiness	Scale-up, solvent removal, sterilization, fiber uniformity	[139]
Surface-tethered systems	Relevant for antimicrobial coatings and device biofilm prevention	Application-specific readiness	Peptide orientation, coating durability, limited diffusion	[144]

**Table 3:** Evidence hierarchy for peptide-based cutaneous anti-infective and wound-healing systems.

System/Formulation	Biological Target	Evidence Level	Model/Study Type	Key Finding	Translational Status	Ref.
Native plant antimicrobial peptides	Bacteria, fungi, microbial membranes	In vitro	MIC, antifungal, membrane assays	Antimicrobial activity reported	Discovery-stage; skin validation limited	[32]
Plant peptide scaffolds: cyclotides, defensins, knottins	Stability, membrane activity, peptide engineering	In vitro/mechanistic	Structural and activity studies	Stable scaffolds for peptide design	Promising templates; clinical use unproven	[23]
AMP-loaded hydrogels	Wound infection and inflammation	Advanced preclinical	In vitro, mouse, porcine wound models	Reduced bacterial burden and inflammation	Strong benchmark; not plant-derived	[100]
Dendritic nanogel–poloxamer AMP system	*Staphylococcus aureus* skin infection	Advanced preclinical	EpiDerm, pig skin, mouse infection models	Improved activity versus free peptide	Topical delivery benchmark; non-plant AMP	[188]
Nanocarrier-based AMP systems	Peptide stability, release, bacterial infection	Mainly preclinical	Nanoparticle characterization, antimicrobial assays	Improved stability and controlled release	Promising; scale-up and toxicity remain barriers	[189]
Lipid nanoparticle AMP systems	Skin infection, peptide protection	Mainly preclinical	Lipid-carrier design and antimicrobial studies	May improve AMP stability and dermal delivery	Experimental; clinical evidence limited	[190]
Electrospun peptide dressings	Local wound delivery, bacterial inhibition	Preclinical	Fiber fabrication, release, antimicrobial assays	Enable local delivery and sustained release	Experimental; scale-up and sterilization unresolved	[191]
Topical pexiganan cream	Mild infected diabetic foot ulcers	Human clinical	Randomized, double-blind trials	One trial failed equivalence; comparable outcomes to ofloxacin	Clinically tested topical AMP; not plant-derived	[186]
Topical omiganan	Atopic dermatitis/seborrheic dermatitis dysbiosis	Human clinical	Randomized phase II and proof-of-concept trials	Safe; microbiome effects observed, clinical efficacy inconsistent	Clinically tested; efficacy not established	[187,192]
Plant-derived peptide–polymer systems for skin therapy	Infection, biofilms, inflammation, repair	Conceptual/early preclinical	Integrated formulation studies remain sparse	Direct clinical evidence is lacking	Requires standardized preclinical and clinical validation	[18,193]

## Data Availability

No new data were created or analyzed in this study. Data sharing is not applicable.
